# Using Test Question Templates to teach physiology core concepts

**DOI:** 10.1152/advan.00024.2022

**Published:** 2023-01-26

**Authors:** Gregory J. Crowther, Thomas A. Knight

**Affiliations:** ^1^Life Sciences Department, Everett Community College, Everett, Washington, United States; ^2^Division of Biological Sciences, University of Washington Bothell, Bothell, Washington, United States; ^3^Department of Biology, Whitman College, Walla Walla, Washington, United States

**Keywords:** conceptual ideas, critical components, exams, formative assessment, summative assessment

## Abstract

The past ∼15 years have seen increasing interest in defining disciplinary core concepts. Within the field of physiology, Michael, McFarland, Modell, and colleagues have published studies that defined physiology core concepts and have elaborated many of these as detailed conceptual frameworks. With such helpful definitions now in place, attention is turning to the related issue of how to maximize student understanding of the core concepts by linking these “big ideas” to concrete student-facing resources for active learning and assessment. Our practitioner-based view begins with the recognition that in many if not most undergraduate physiology courses assessment drives learning. We have therefore linked published conceptual frameworks to Test Question Templates (TQTs), whose structure promotes transparent assessments as well as the active learning needed to prepare for such assessments. We provide examples of conceptual framework-linked TQTs for the physiology core concepts of Homeostasis, Flow Down Gradients, the Cell Membrane, and Cell-Cell Communication. We argue that this deployment of TQTs has at least two distinct benefits for the teaching and learning of core concepts. First, documenting the connections between conceptual frameworks and TQTs may clarify coverage and assessment of the core concepts for both instructors and students. Second, misconceptions about core concepts may be directly targeted and dispelled via thoughtful construction, arrangement, and iteration of TQTs. We propose that the TQT framework or similar approaches may be applied fruitfully to any sufficiently articulated physiology core concept for high school, undergraduate, or graduate students.

**NEW & NOTEWORTHY** Our students often focus on the grades they need to advance through academic programs. How can instructors harness this understandable interest in grades to help students gain a true understanding of core concepts? The new framework of Test Question Templates (TQTs) shows promise in linking student priorities like test scores to instructor priorities like core concepts.

## A CORE CONCEPT DREAM/NIGHTMARE

Your undergraduate physiology students are starting a midterm exam. The first question is this: “How is the control of plasma glucose levels similar to the control of plasma calcium levels?” You are eager to see how students respond, since when you covered these two examples of homeostasis in lecture you were careful to use consistent terminology, referring in both cases to set points, sensors, control centers, effectors, and negative feedback.

*Student A* thinks, “This is pretty straightforward. Both plasma calcium levels and plasma glucose levels are homeostatically regulated variables, meaning that they require set points, chemoreceptor sensors to sense the current concentrations, control centers to compare current levels to the set point and send out endocrine responses, and effectors to reduce discrepancies between the current levels and the set point. This reduction of discrepancies is called negative feedback in both cases.”

*Student B*, however, is not quite as clear on the point of the question. “Well,” they think, “we have hormones to control both plasma calcium and plasma glucose. But the specific hormones are different and are made by different glands, and the responses to the hormones are different, too. Maybe I should just say that the endocrine system handles both?”

Meanwhile, *student C* is drawing a blank until eventually coming up with the word “feedback.” “They both involve feedback, maybe?” they think. “There were two types of feedback, weren’t there? Like, um, afferent and efferent? No, that was something else. I think it was positive and negative. Negative sounds bad, so it’s probably positive. Let’s go with ‘both involve positive feedback to keep levels balanced.’”

We offer this hypothetical scenario to acknowledge that we have all been in similar situations, ones where we thought we had taught core concept ideas with admirable clarity and emphasis, yet many students’ test performances suggest otherwise. How can we all do better?

## TEACHING AND LEARNING CORE CONCEPTS: HOW DOES ASSESSMENT FIT IN?

In teaching a subject like biology, certain “big ideas,” or core concepts, are considered central to the discipline. For undergraduate biology, the Vision and Change report (1) proposed five core concepts: evolution; structure and function; information flow, exchange, and storage; pathways and transformations of energy and matter; and systems. These five core concepts were subsequently unpacked into component principles and specific statements in the BioCore Guide (2). The five core concepts were distinguished from six core competencies, or skills, also outlined initially by Vision and Change (1) and later unpacked in the BioSkills Guide (3). Meanwhile, a team of physiology educators led by Joel Michael, Jenny McFarland, and Harold Modell developed a set of physiology-specific core concepts (4–6) and have unpacked several of these into conceptual frameworks of component statements and terms (5, 7–11). In both the general biology- and physiology-specific projects, there were multiple rounds of input from diverse faculty, offering assurance that the final core concept definitions are not simply the opinions of the authors but instead reflect broad consensus within the biology and physiology education communities.

Any instructor who focuses on core concepts must consider the question of how to use assessments to determine whether students understand the core concepts being taught. Since defining the core concepts and unpacking them are herculean tasks (1, 2, 5), it is understandable that these projects did not initially grapple with practical assessment issues in detail (though a homeostasis concept inventory was eventually created; Ref. 12). Moreover, thoughtful assessment in any cognitively rich biology course is inherently complicated. A straightforward one-to-one mapping of core concepts to assessment items is not necessarily feasible or desirable, since an ideal summative assessment would presumably cover core concepts, core competencies, and “noncore” material. However, in our view, any practical department- or course-level initiative to help students learn core concepts (and/or core competencies) must carefully consider assessment strategies, because, from the students’ perspective, “assessment drives learning” (13, 14), i.e., students mostly study what they think will be on the summative assessments. This assertion, if accepted, arguably has at least two important implications:

- Instructors should assess students on what we really want them to learn. In other words, we need strong alignment between learning objectives (LOs) and assessments.
- Instructors should help students learn, practice, and review the material in ways that connect transparently to assessments. In other words, we also need strong alignment between learning activities and assessments (what some might call “teaching to the test”).

Our broad impression of undergraduate biology education in the United States is that *implication A* is more widely accepted and practiced than *implication B*. As an example, consider the now-common practice of “Blooming” assessment questions according to the cognitive hierarchy originally proposed by Benjamin Bloom and colleagues (15–19). Many instructors “Bloom” their assessments because they want to see whether they are assessing the higher-order cognition that is reflected in their LOs. We applaud this practice but consider it insufficient since, in and of itself, it does not address *implication B*. In other words, using test questions that require mastery of important LOs is important but does not necessarily mean that students are able to practice those LOs in the ways required by the tests.

Regarding *implication B*, diverse opinions on teaching to the test (20, 21) suggest that not all educators share our enthusiasm for closely aligning activities and assessments. However, there is evidence that adding active-learning practice on short-answer questions styled in the format of actual exam questions improves introductory biology students’ test performance (22) and reduces the so-called achievement gap between historically underserved students and other students (23). Similar approaches have been implemented with apparent success in undergraduate anatomy and physiology courses (24, 25).

We believe that the recently introduced framework of Test Question Templates, or TQTs (26, 27), is especially well suited for simultaneously addressing *implication A* and *implication B*, both in general and in regard to core concepts in particular. In the rest of this article, we first review TQTs and then illustrate how students’ understanding of physiology core concepts may be enhanced via the careful coupling of conceptual frameworks and TQTs.

## WHAT IS A TQT?

A TQT is a student-facing resource (i.e., a resource that is visible to and written for students) that explicitly connects a lesson learning objective (LLO) with multiple specific examples of how that LLO might be assessed on a test.

**Figure 1.**
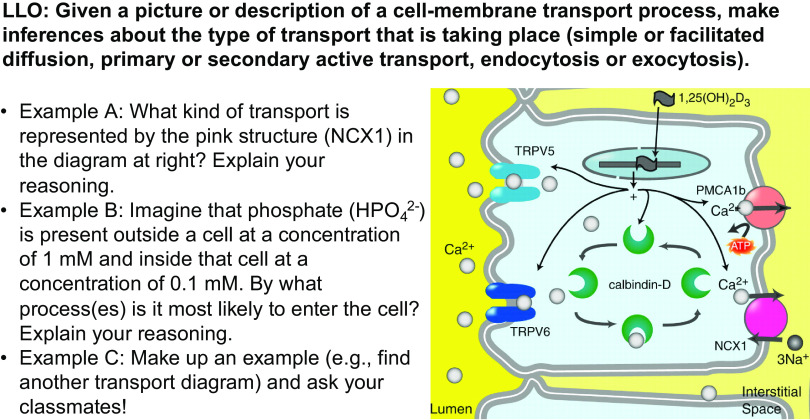
A Test Question Template (TQT) aligned with the physiology core concept of the Cell Membrane. LLO, lesson learning objective. Diagram taken from Ref. 28, with permission from the American Physiological Society.

A TQT begins with a statement of a LLO (more specific than a course-wide learning objective), sometimes called an input-output statement because it is of the form of “given X, students will do Y” (Fig. 1). The TQT then provides multiple example questions showing how the LLO can be examined, practiced, and met/achieved, as well as how it could conceivably be assessed on the subsequent summative assessment (test). The examples are fairly different from each other to hint at the range of possible questions while still conforming to the input-output statement. In Fig. 1, for instance, *example A* involves interpreting an image whereas *example B* is entirely text based, yet both examples are rooted in the LLO of inferring transport mechanisms from the information given.

Perhaps the single most important feature of a good TQT is that its LLO is specific enough to focus attention on particular concepts or skills yet broad enough to allow for a large number of example questions. Its structure should allow instructors to readily create novel questions (i.e., questions that students have not seen before) that, via their novelty, probe students’ true understanding of the LLO (rather than their memorization ability or test-taking savvy). Instructors can thus use TQTs to give students valuable guidance on the scope and format of upcoming test questions without giving away the exact details of the questions.

Since students are often very focused on how they will be assessed—a rational, reasonable stance—we have found (unpublished observations) that students are most invested in and appreciative of TQTs when instructors use TQTs consistently throughout a course to show students what will be expected of them on tests. For example, one of us (G.J.C.) tells students that short-answer questions will comprise a majority of the points on every test and that every short-answer question will directly reflect a previously practiced TQT. This messaging clearly conveys that TQTs cover the most important, most testworthy, most studyworthy material. Moreover, it encourages some students to think of additional example questions (see Fig. 1, *example C*) as further exploration of the various ways in which an LLO may be understood and assessed.

## TQT *BENEFIT #1*: DESIGNING AND REVISING CORE-CONCEPT COVERAGE

The leaders of the core concept movements have consistently acknowledged that individual departments and instructors must choose which core concepts should be covered in which individual courses. Once those decisions are made, how might instructors determine whether they are focusing on their chosen core concepts effectively?

We propose two basic goals for core concept coverage in a course: first, that a core concept be explored via examination of specific subconcepts of that core concept and, second, that these examinations occur repeatedly during a course. The first goal hinges on the fact that, as summarized in Table 1, each core concept is made up of subconcepts that can be arranged in a multilevel hierarchical structure (5, 6). Therefore the first goal is that a course should cover the specific subconcepts that are essential to the chosen core concept in the context of the course. The second goal is that, for optimal reinforcement of each chosen core concept and its subconcepts, these should be taught at multiple times during the course (e.g., when exploring different organ systems).

**Table 1.** An overview of conceptual framework subconcepts referenced in this article

Core Concept	Critical Components, Constituent Ideas, and Elaborations	Reference
Homeostasis	Critical Components H1 (includes Constituent Ideas H1.3–1.5), H2 (includes Constituent Ideas H2.1–2.3), H3 (includes Constituent Ideas H3.3 and H3.4), H4, and H5	(7)
Flow Down Gradients	Critical Components F1, F2, F3, F4 (includes Constituent Ideas F4.1 and F4.2), and F5	(5)
Cell Membrane	Critical Components CM1 and CM2 [includes Constituent Ideas CM2.1, CM2.2 (includes Elaborations CM2.2.1 and CM2.2.2), CM2.3, CM2.4 (includes Elaboration 2.4.1), CM2.5]	(9)
Cell-Cell Communication	Critical Components CC1, CC2, CC3, CC4, CC5 (includes Constituent Idea CC5.1), CC6, and CC7	(8)

In this article, we use the term “subconcepts” to refer generally to the pieces of a core-concept hierarchy without specifying a particular hierarchical level. Michael and McFarland (6) have previously defined Critical Components, Constituent Ideas, and Elaborations as increasingly fine-grained pieces of a core concept’s conceptual framework. For example, the conceptual framework of the cell membrane (9) includes Critical Component CM2 (“the cell membrane participates in a variety of mechanisms that maintain the integrity of cells and make possible the specialized function of any cell”), which encompasses several smaller Constituent Ideas such as CM2.2 (“the cell membrane helps to determine solute concentrations inside the cell to preserve the viability of the cell and the continuation of its specialized functions”), which in turn encompasses several even smaller Elaborations such as CM2.2.1 [“lipid soluble molecules (O_2_, CO_2_, urea) can passively diffuse across the membrane through the lipid portion of the membrane”].

One could imagine various audits by which educators could check whether they are meeting these two goals. For example, an instructor could review a course’s lecture slides and notes and check off the points at which a given core concept and its key subconcepts were taught. This exercise could certainly be useful, but the fact that an instructor has presented a core concept and discussed it with appropriate context does not guarantee that students understand it well enough to apply it to a novel assessment question. If instead we embed TQTs throughout a course, encourage the students to actively practice identified core concepts via these TQTs, and then use these TQTs to write test questions (as recommended above), an audit of the TQTs may be especially informative and meaningful. That is, if a core concept’s key subconcepts appear in TQTs in multiple units of the course, the core concept is likely being covered in a manner that is thorough, transparent, and memorable to students.

To illustrate the potential value of this approach, we present a self-audit of a 200-level human physiology course taught by one of us (G.J.C.). This survey course, which marches through the body’s organ systems in one 10-wk quarter, is taken mostly by pre-nursing and other pre-allied health students after they have fulfilled prerequisites of one-quarter courses in general chemistry, cell and molecular biology, and human anatomy. The course is designed to emphasize the core concepts of Cell-Cell Communication, the Cell Membrane, Flow Down Gradients, and Homeostasis. An audit of the 130 TQTs included in the Spring 2021 course suggested that certain core concepts and subconcepts were included successfully (Table 2). For example, the core concept of Flow Down Gradients was highlighted in TQTs for the integumentary, nervous, cardiovascular, respiratory, and reproductive units, and subconcepts F2, F3, F4, and F5 were all addressed with TQTs in multiple organ systems.

**Table 2.** Core concepts and subconcepts in a 200-level human physiology course

Unit/System	Homeostasis	Flow Down Gradients	Cell Membrane	Cell-Cell Communication
Introduction	H2–H5	F2, F4	CM2.2	
Integumentary		F5		
Muscular				
Nervous		F4, F5	CM2.2, CM2.3	CC1, CC3–CC5
Endocrine			CM2.2, CM2.3	CC4, CC5
Cardiovascular	H4, H5	F2–F5		CC7
Immune			CM2.5	
Respiratory		F2		
Digestive			CM2.2	
Urinary	H3–H5			
Reproductive		F3, F5		CC5

The leftmost column lists the units/organ systems examined in this course. A core concept is named at the top of each of the 4 remaining columns, and each cell of the table indicates the subconcept(s) of these core concepts taught using Test Question Templates (TQTs) for that unit (row) in Spring 2021. For example, as shown here, the Introduction unit included TQTs addressing the core concept of homeostasis (specifically about Critical Components H2 through H5 but not Critical Component H1). Subconcept abbreviations (e.g., H1, F2, CM2.2) are as presented in the published conceptual frameworks (Refs. 5, 7–9; see Table 1). The Muscular unit lessons did not include any subconcepts from these 4 core concepts but did include TQTs addressing the core concept of Structure-Function (not examined in this article).

The self-audit also revealed gaps or missed opportunities for teaching these core concepts. Here is one example: the high-priority core concept of Cell-Cell Communication includes seven critical components, CC1–CC7 (8), but CC2 (“Transport of messenger molecules is determined by the chemical nature of the messenger”) and CC6 (“Termination of a messenger signal is accomplished in several ways”) were not directly addressed by any TQT in the course (Table 2). The audit thus helped the instructor notice this gap in his coverage of Cell-Cell Communication and consider possible remedies. A second example of a gap was a failure to reinforce core concepts and/or subconcepts in additional units where they would have fit in well; e.g., the respiratory unit did not include any TQTs on Homeostasis, despite the respiratory system’s importance in keeping plasma CO_2_, O_2_, and pH levels near their set points. This observation led the instructor to write a new respiratory system TQT to cover this gap. One might also note that the muscular unit did not include TQTs addressing any of the four emphasized core concepts (though TQTs addressing the core concept of Structure-Function were included) and that perhaps one or more should be added (e.g., on cell-cell communication at the neuromuscular junction). Thus, a TQT-focused audit may suggest possible strengths and limitations in one’s current coverage of chosen core concepts. We stress that we have no specific beliefs about how many core concepts should be covered how many times during a course; we present this particular audit only as an example of how pedagogical intentions could be checked against pedagogical execution.

Beyond the basic goal of iterative exploration of each targeted core concept’s key subconcepts, instructors should consider the additional goal of helping students understand how the subconcepts contribute to the core concept as a whole. Put another way, how might we reconcile core concepts’ inherent broadness with our need for more granular formative and summative assessment items? As we see it, the broad concepts and narrower assessment items must be arranged into clear, explicit hierarchies that will probably resemble the conceptual frameworks themselves. An example of this approach in a physiology-related course can be seen in Physiological Ecology (Biology 403) as taught by Deborah Donovan at Western Washington University (personal communication). In student-facing materials, Donovan connects each of her course’s success criteria (similar to the LLOs of TQTs) to a broader learning target and each learning target to a still-broader core concept. For example, success criteria like “I can analyze a graph comparing the metabolic rate of a homeothermic endotherm to ambient temperature” and “I can predict how and explain why the metabolism-temperature relationship of a homeothermic endotherm will change during different seasons” are shown to serve a learning target about homeothermic endotherms, which in turn is one component of a core concept about the control of body temperature.

**Figure 2.**
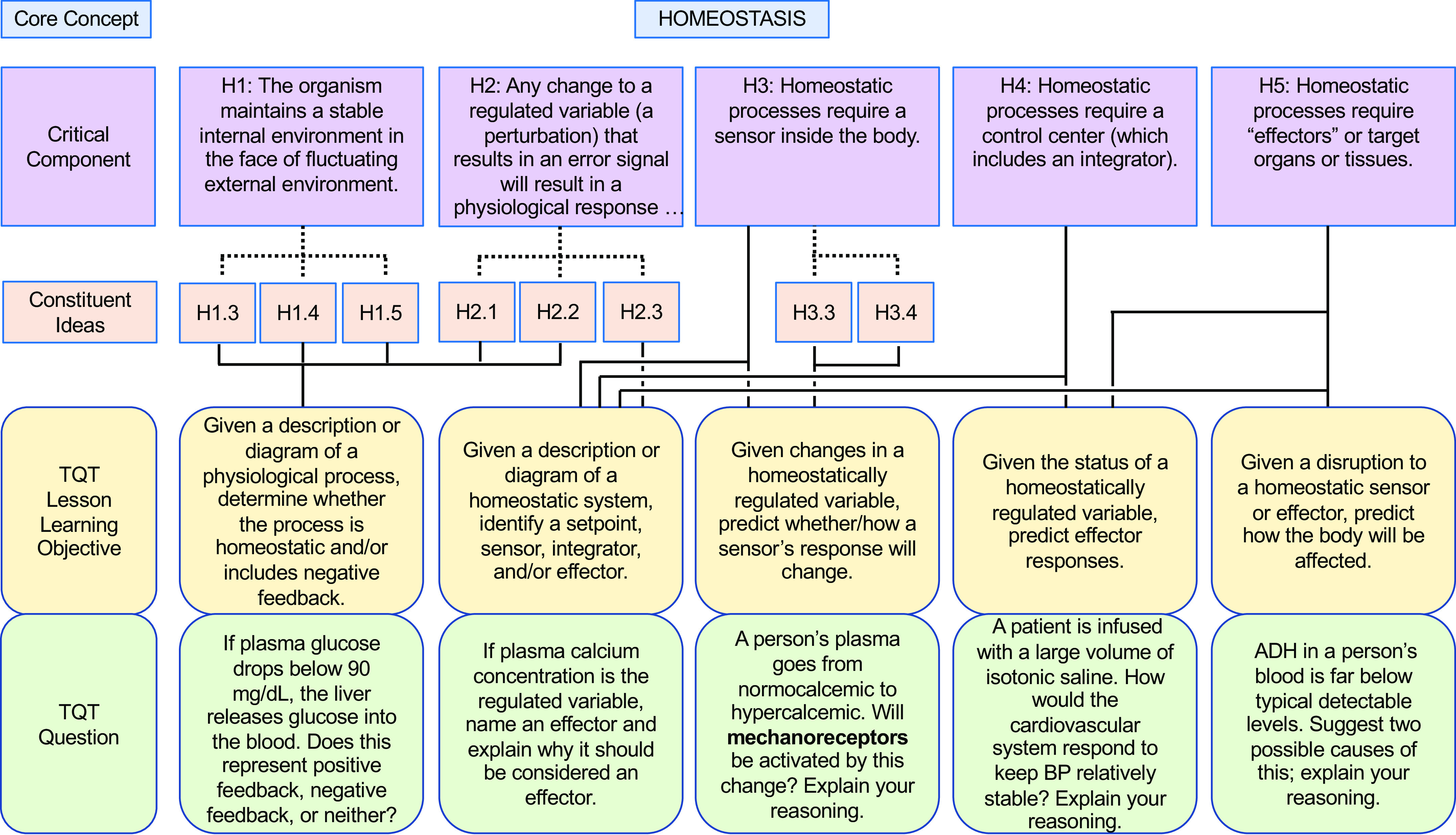
An example of how the core concept of Homeostasis could be covered via conceptual framework-linked TQTs. Subconcepts are as in the Homeostasis conceptual framework of Ref. 7 (see also Table 1); for clarity, we show only the subconcepts (Critical Components and Constituent Ideas) directly related to the TQTs. Each TQT is composed of a lesson learning objective (LLO) and multiple specific classroom/assessment questions, of which only 1 is displayed here (*bottom* row) because of space constraints. Dotted lines indicate relations within core concept hierarchy (see Table 1); solid lines indicate relations of TQTs to that hierarchy. A more focused view of H3 (H3.3 and H3.4) and its aligned TQT is shown in Fig. 6.

**Figure 3.**
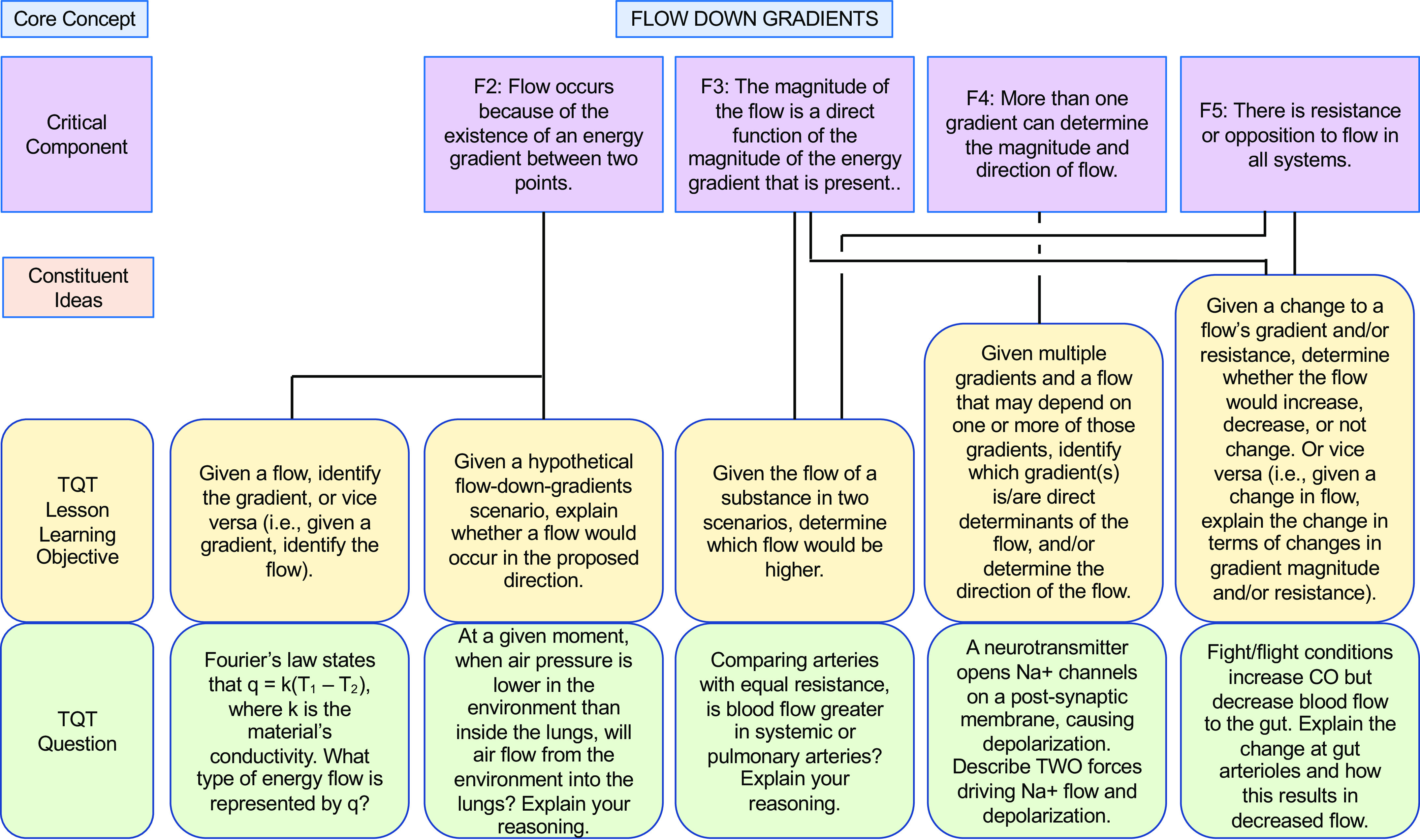
An example of how the core concept of Flow Down Gradients could be covered via conceptual framework-linked TQTs. Subconcepts are as in the conceptual framework of Michael et al. (5). Conventions are as in Fig. 2. A more focused view of F4 and its aligned TQT (2nd from *right*) is shown in Fig. 7.

**Figure 4.**
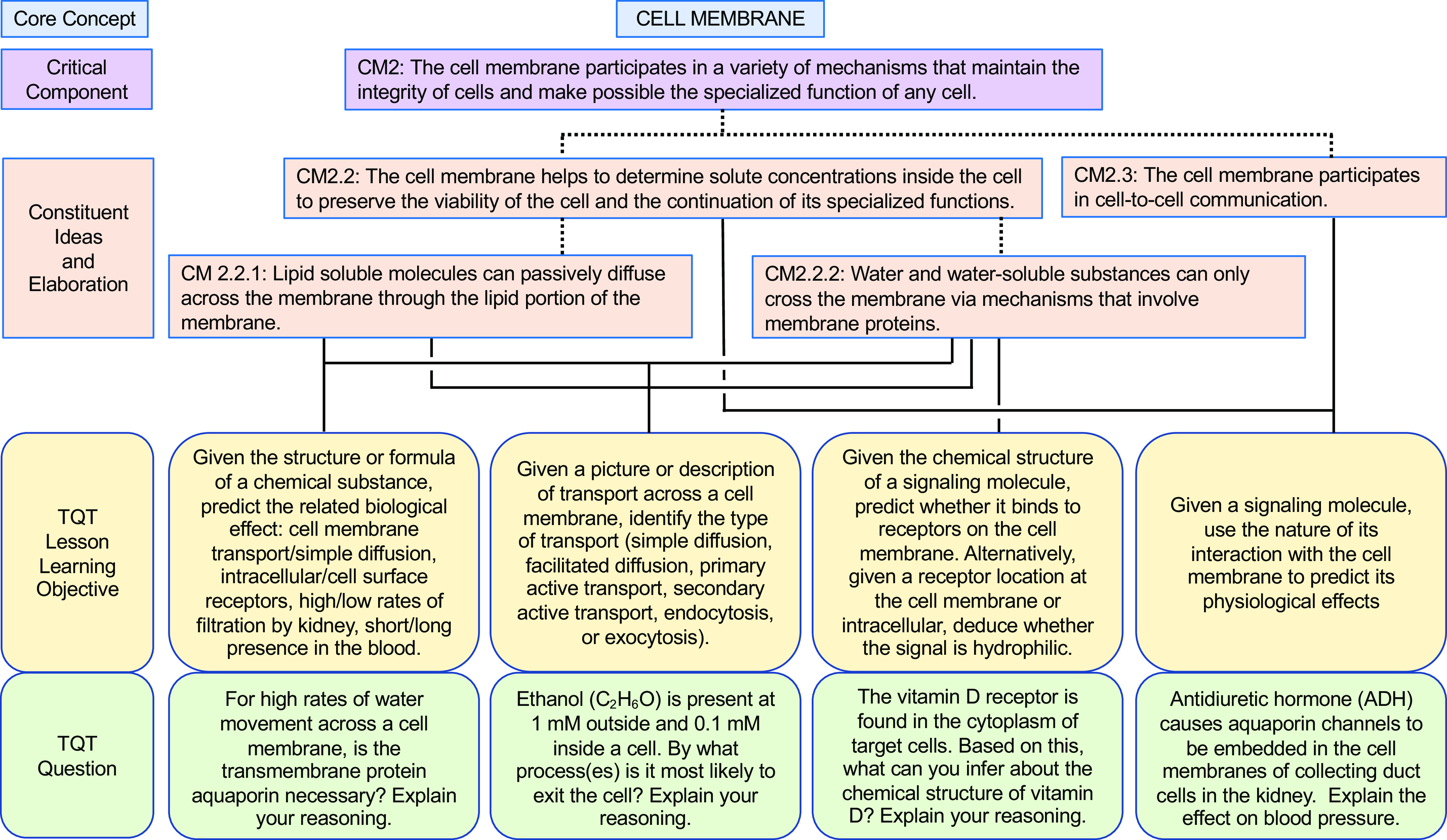
An example of how the core concept of Cell Membrane could be covered via conceptual framework-linked TQTs. Subconcepts are as in the conceptual framework of Michael and Modell (9). Conventions are as in Fig. 2. A more focused view of CM2 (CM2.2.1 and CM2.2.2) and its aligned TQT (*bottom left*) is shown in Fig. 8.

**Figure 5.**
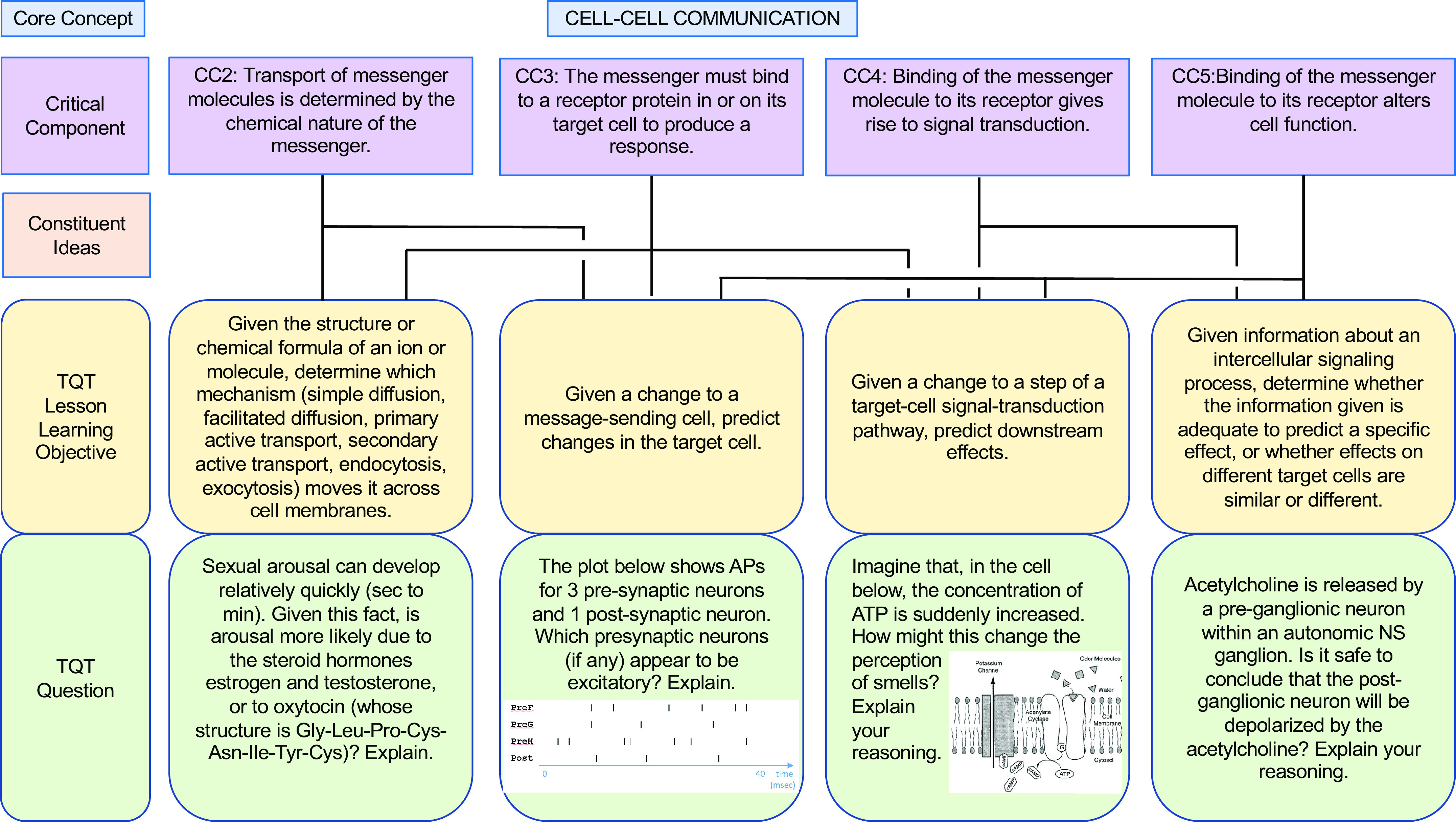
An example of how the core concept of Cell-Cell Communication could be covered via conceptual framework-linked TQTs. Subconcepts are as in the conceptual framework of Michael et al. (8). Conventions are as in Fig. 2. A more focused view of CC5 (and CC5.1, not shown) and its aligned TQT (*bottom right*) is shown in Fig. 9. Image of olfactory transduction taken from Ref. 29, with permission from the American Physiological Society.

In our own similar efforts to more deliberately align our teaching with core concepts (and subconcepts) and help students appreciate the interconnectivity, we have forged connections between key subconcepts and TQTs, thus translating these subconcepts into concrete student-facing questions for active learning and assessment. To illustrate our alignment and examples of specific questions, we present examples of TQTs aligned with conceptual framework subconcepts for Homeostasis, Flow Down Gradients, Cell Membrane, and Cell-Cell Communication (Figs. 2, 3, 4, and 5, respectively).

As we developed TQTs, the direct inspiration for most came from lecture material rather than from published core concepts and conceptual frameworks, with TQTs subsequently linked to core concepts when auditing and designing subsequent courses (e.g., Table 2). An advantage of this approach is that the TQTs fit naturally into the lecture material and could be seen by students as a review and direct extension of the lecture/text material. This approach has had two additional consequences. First, since TQTs were not initially written to cover specific subconcepts, most TQTs relate to more than one subconcept, with some connecting to as many as four or five [Figs. 2–5; a given TQT LLO may receive connections from multiple subconcepts (solid lines)]. Second, each set of TQTs need not encompass the entirety of a core concept, as is evident in Figs. 2–5 (e.g., see Fig. 3, where subconcepts that connect directly to a TQT are shown; other subconcepts, like F1, are omitted). Collectively, these two consequences reflect the fact that we have not sought to create a TQT for every single subconcept; we have instead aimed to create TQTs that are biologically interesting and closely aligned with existing lecture material, while accepting that not all important subconcepts lend themselves to TQTs. We suggest that departments and instructors consider a similar approach based on what they believe their students need most in the time available (i.e., not all subconcepts need to be explicitly explored in a given unit or course).

## TQT *BENEFIT #2*: CORRECTING CORE-CONCEPT MISCONCEPTIONS

Good teaching is, of course, not just a matter of delivering well-organized information; it also involves helping students identify, reflect on, and correct their misconceptions. Misconceptions can be defined as “inaccurate ideas that can predate or emerge from instruction” (p. 5 of Ref. 30). By that definition, misconceptions can be important, unimportant, or anywhere in between. In considering the numerous core-concept misconceptions that students may harbor (5, 31, 32), the conceptual frameworks can help us judge their relative importance. For example, for the conceptual framework of the Cell Membrane (9), an instructor might deem a misconception about tight junctions (CM2.4.1) less important than a misconception about the structure of the cell membrane as a whole (CM1), since the latter subconcept is at a higher level of the framework (i.e., in the language of Ref. 6 and Table 1, CM1 is a Critical Component whereas CM2.4.1 is a lower-tier Elaboration). Indeed, when Michael and Modell (9) asked physiology faculty to rate the relative importance of the Cell Membrane subconcepts, the Critical Components tended to be rated as somewhat more important than their constituent Elaborations (Table 1 of Ref. 9). By this kind of hierarchy-based reasoning, one might also deem a misconception about tight junctions (Elaboration CM2.4.1) less important than a misconception about protein-mediated membrane transport (Elaboration CM2.2.2) based on the fact that the latter Elaboration encompasses six substituents (CM2.2.2.1 through CM2.2.2.6).

We believe that linking these conceptual frameworks to TQTs provides an additional advantage in revealing and dispelling misconceptions. This belief is based on Ambrose and colleagues’ practical recommendations (33) for addressing misconceptions (p. 37–38), which include “ask students to make and test predictions” (since finding that predictions are wrong can be a wake-up call), “ask students to justify their reasoning” (since this may reveal internal contradictions that can then be addressed), and “provide multiple opportunities for students to use accurate knowledge” (since persistent or nuanced misconceptions may need to be counteracted multiple times during exploration of varying topics). These tips could perhaps be condensed into the following general directive: make lots of related practice problems available to students so that they can notice incorrect predictions/explanations, identify their weak points amidst other valid knowledge, and try again on other related problems. This directive is entirely compatible with the TQT framework, in which related problems are explicitly grouped, facilitating the practice of repetition with variation.

**Figure 6.**
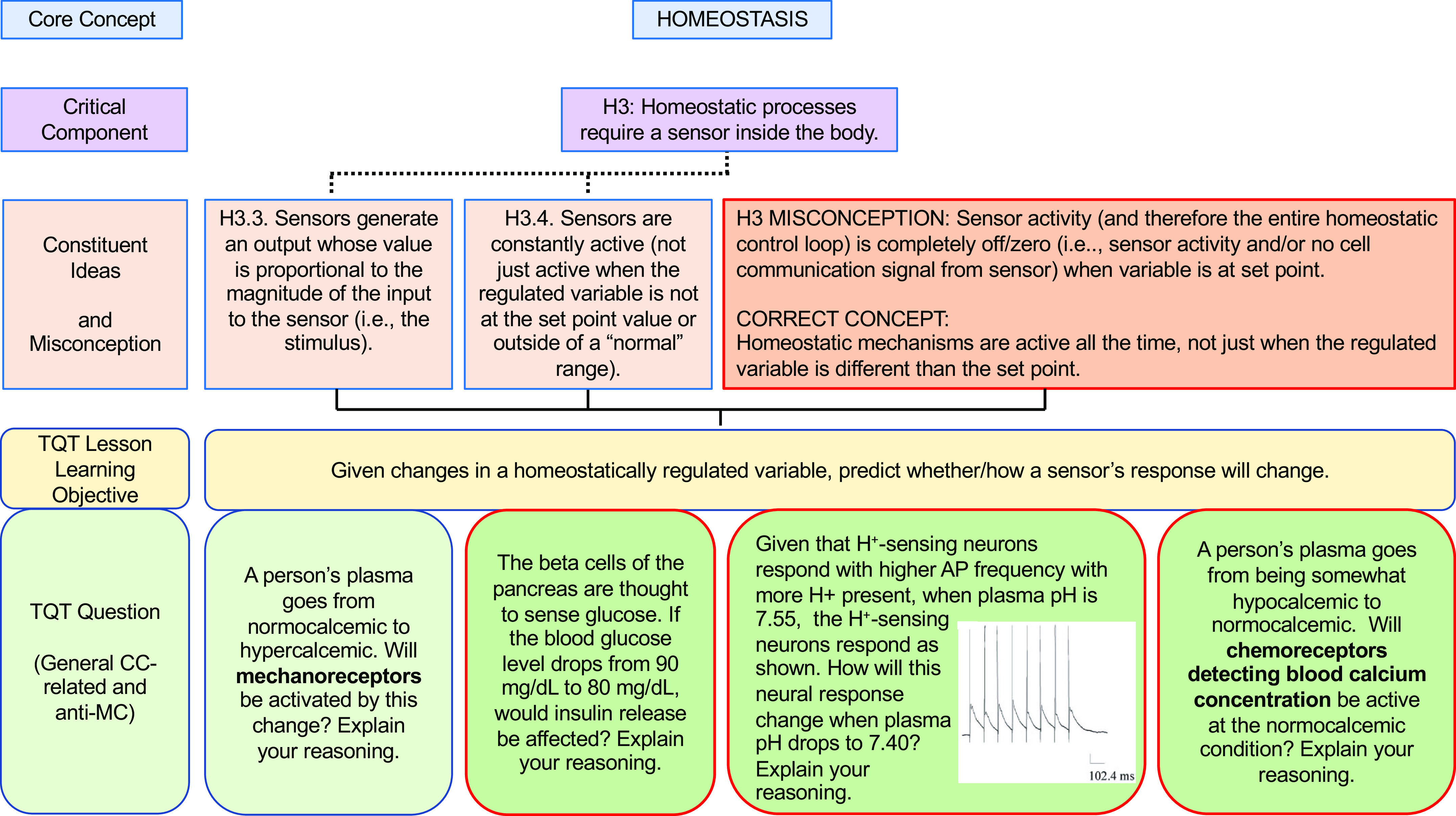
A TQT aligned with the Homeostasis conceptual framework to directly confront a misconception. This TQT lesson learning objective (LLO) examines sensor function (Critical Component H3 and its Constituent Ideas H3.3 and H3.4) and H3-related student misconception (red-framed box). *Bottom* row: TQT questions explore this LLO; the question on *left* examines H3 in general, and the 3 red-framed TQT questions on *right* directly confront the misconception across different organ systems. Subconcepts are as in the Homeostasis conceptual framework of Ref. 7; the misconception is from Ref. 5. Image of action potentials adapted from Ref. 34, with permission from the American Physiological Society.

**Figure 7.**
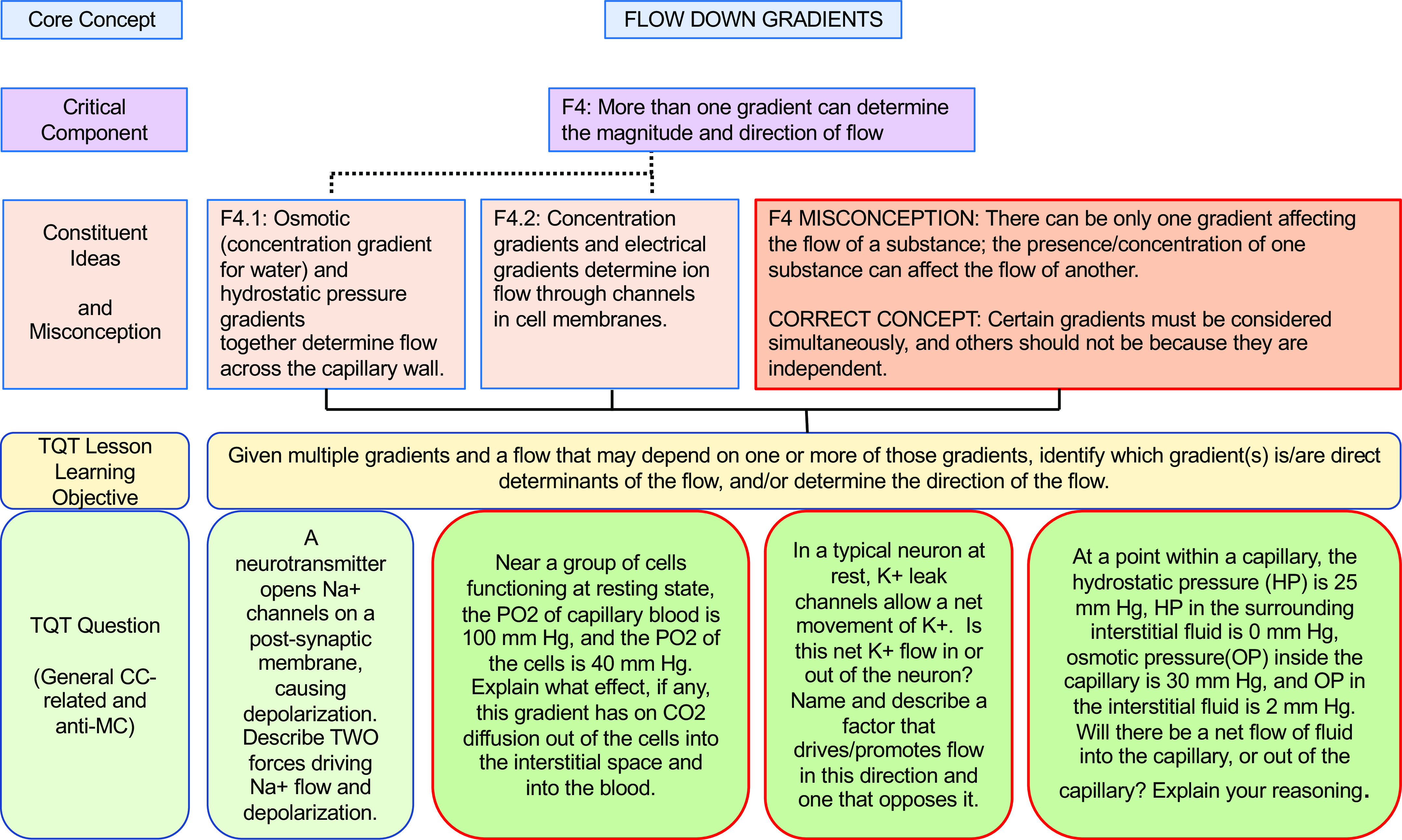
A TQT aligned with the Flow Down Gradients conceptual framework to directly confront a misconception. This TQT lesson learning objective (LLO) examines simultaneous gradients (Critical Component F4 and its Constituent Ideas F4.1 and F4.2) and a F4-related student misconception. Conventions are as in Fig. 6. Subconcepts are as in the Flow Down Gradients conceptual framework; the misconception is from Ref. 5.

**Figure 8.**
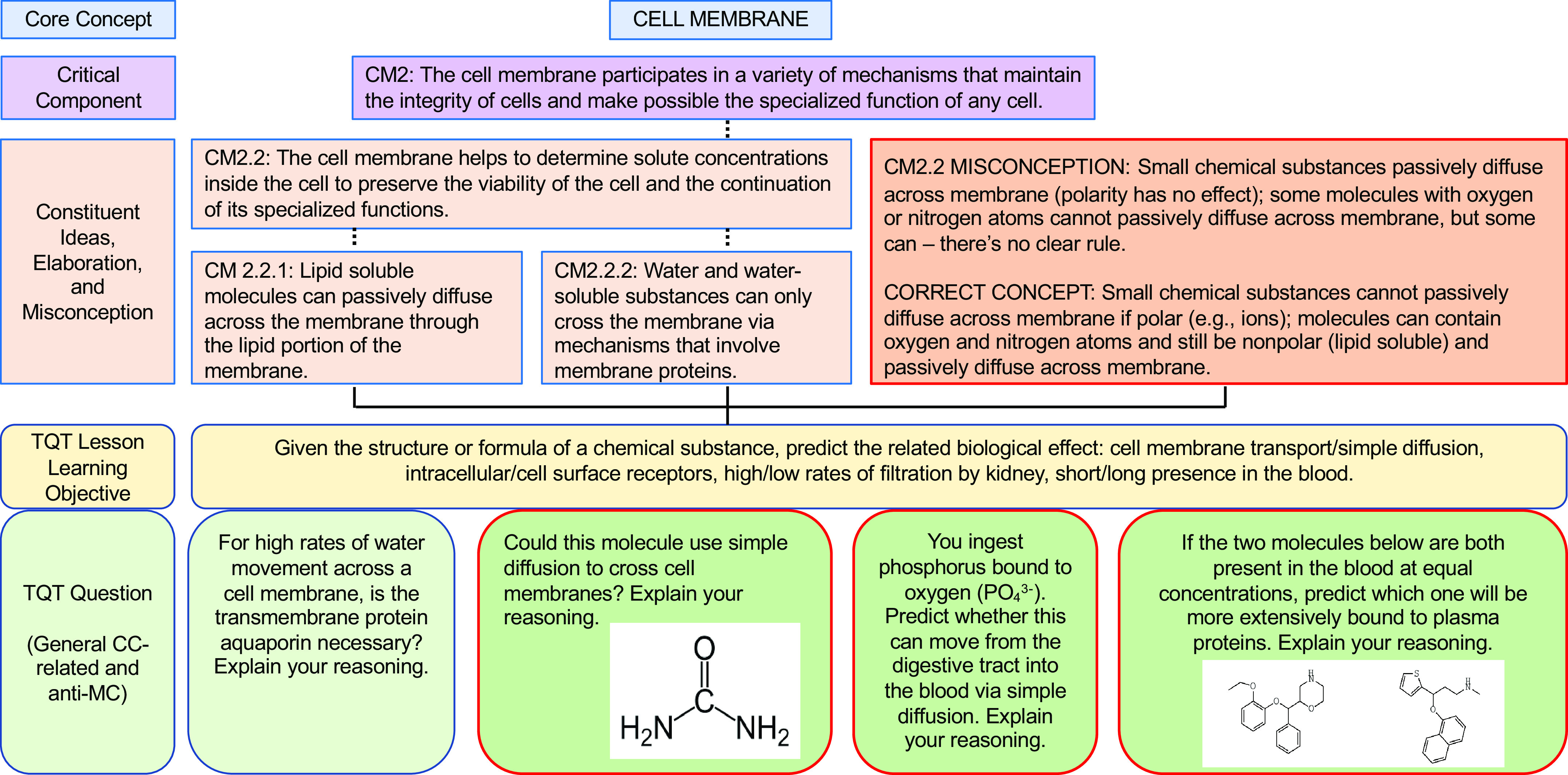
A TQT aligned with the Cell Membrane conceptual framework to directly confront misconception. This TQT lesson learning objective (LLO) examines membrane transport (critical component CM2.2 and its constituent ideas CM2.2.1 and CM2.2.2) and CM2.2-related student misconception. Conventions are as in Fig. 6. Subconcepts are as in the cell membrane conceptual framework of Michael and Modell (9).

**Figure 9.**
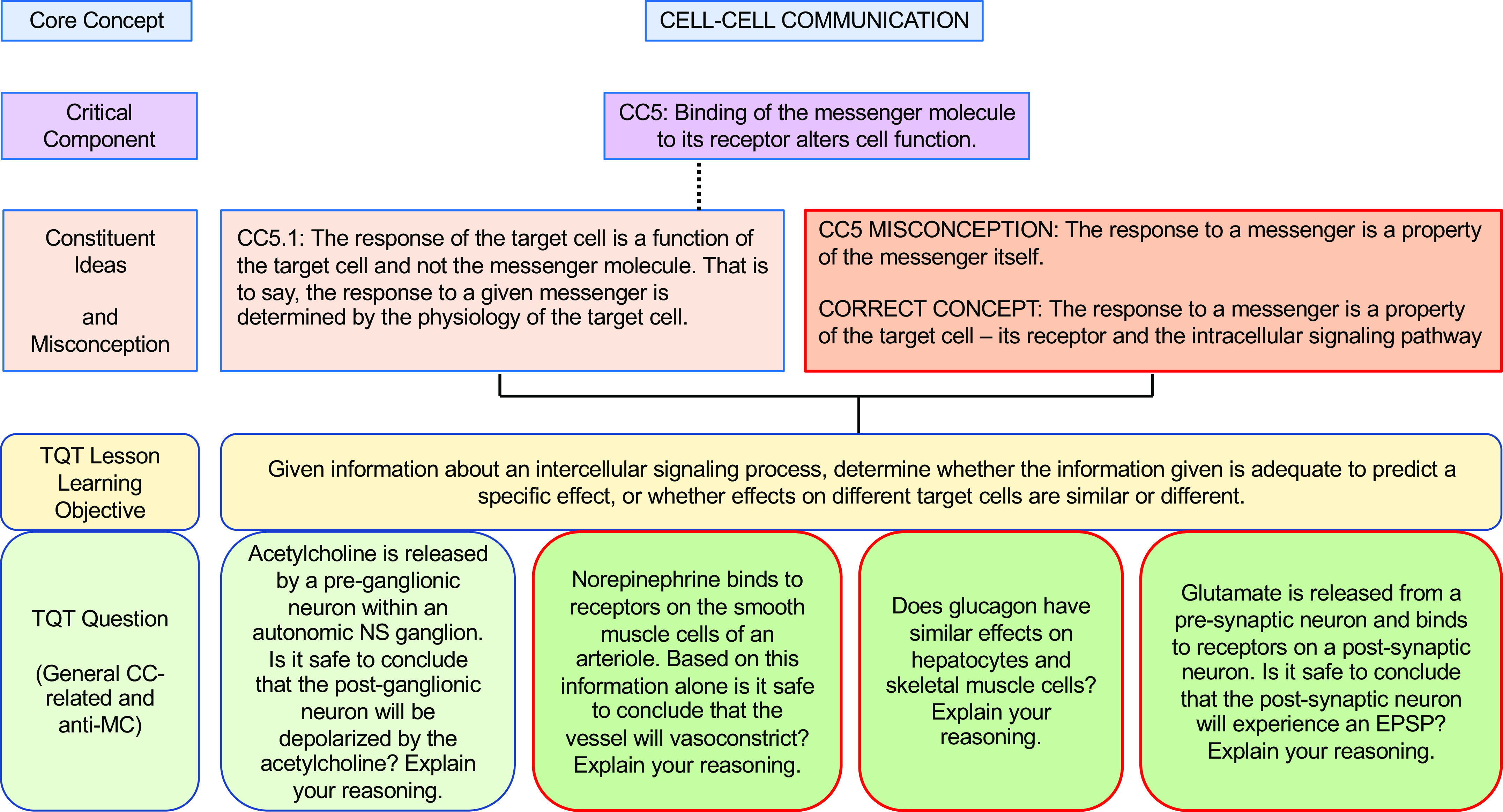
A TQT aligned with the Cell-Cell Communication conceptual framework to directly confront a misconception. This TQT LLO examines the response of a target cell (Critical Component CC5 and its Constituent Idea CC5.1) and CC5-related student misconception. Conventions are as in Fig. 6. Subconcepts are as in the Cell-Cell Communication conceptual framework; the misconception is from Michael et al. (8).

We thus propose that TQTs can be a useful tool for unmasking misconceptions and helping students overcome them. To this end, we provide examples that extend the connections between conceptual framework TQTs to more directly address misconceptions, one for each of the core concepts of Homeostasis, Flow Down Gradients, the Cell Membrane, and Cell-Cell Communication (Figs. 6, 7, 8, and 9, respectively). Although these examples are not prescriptive, they illustrate the general strategy of employing TQTs that are broad enough to be reused across multiple organ systems yet specific enough to focus attention on the misconception and closely related issues. Let us take Fig. 6 as an example, which tackles the common misconception that homeostatic mechanisms are active only when the regulated variable diverges from its set point. The four TQT questions in Fig. 6 focus on sensors involved in homeostatic feedback, examining the regulation of plasma calcium (usually covered in skeletal and/or endocrine units), plasma pH (usually covered in cardiovascular, respiratory, and/or urinary units), and plasma glucose (usually covered in endocrine and/or digestive units). The first of the four TQT questions addresses the core concept (and Critical Component H3) in general, whereas the following three TQT questions (see Fig. 6, *bottom* row) directly poke at the misconception (see “H3 Misconception” and “Correct Concept” in Fig. 6 box), challenging students’ understanding so that any misconceptions can be revealed, defined, and revised into a more correct understanding. In this figure and the others (Figs. 7–9), all four bottom-row questions (and alternatives not shown) align with a single TQT LLO that in turn is aligned with the conceptual framework, yet the LLO is general enough to be used across these example questions, across different organ systems, and even across courses (e.g., A&P I to A&P II).

As we have seen, the repetition with variation recommended by Ambrose and colleagues (33) is intentionally built into Figs. 6–9. Is such repetition truly necessary, since it leaves less time for completely new material? Our response is rooted in Vision and Change’s observation (1) that biology courses have historically been tilted too far toward breadth at the expense of in-depth understanding of the core concepts, and this repetition with variation offers a partial corrective. Among other benefits, repetition (especially with different specifics from different topics/organ systems) increases the likelihood that an understanding of these core concepts will stay with students well beyond the end of a particular course. In addition to providing needed iterative practice and assessment, TQTs’ explorations of the same core concept and subconcepts in different contexts (e.g., different organ systems) help students appreciate the pervasiveness of the core concepts throughout biology and physiology. To put it another way, if students only learn sensor function in thermoregulation, they might retain misconceptions about sensors that hinder a full, general understanding of sensors in homeostatic systems.

## EQUITABLE TEACHING OF CORE CONCEPTS

The previous paragraph can also be viewed through the lens of equitable teaching, i.e., structuring classes to put success within the reach of as many students as possible (35). As suggested by our opening vignette, most classes include individuals who vary widely in initial content knowledge, study skills, self-efficacy, time available for studying, and so on. When TQTs are implemented as suggested above, they give students multiple opportunities to dispel misconceptions and acquire and demonstrate competence, so that initial struggles and occasional setbacks do not preclude eventual acquisition and demonstration of mastery (36).

Our opening vignette also implies that physiology instructors typically assess students via traditional high-stakes exams, and, indeed, TQTs were originally invented to help students prepare for such exams (26). However, in light of well-reasoned critiques of and improvements upon traditional grading systems (36–40), TQTs can also support alternative grading systems that include tests, such as specifications (specs) grading (37) and standards-based grading (36). In these approaches, student work is graded according to whether it meets an expected standard or specification (“spec”) of competence or mastery. Importantly, students whose work initially falls short of the standard are generally given additional chances to meet the standard, which generally necessitates creating additional versions of each test, which can be a significant burden on instructors. However, this burden can be greatly eased if tests are based directly on well-constructed TQTs. Imagine, for example, that students need to pass a “homeostasis standard” by correctly answering four out of five questions about homeostasis on a test. If each test question is based on a separate well-structured TQT, it should be feasible to spin off numerous examples from each TQT to create several versions of the test, all roughly equal in difficulty (41).

Overall, then, by facilitating multiple rounds of core concept usage both in studying and on tests, TQTs can help make the teaching of physiology core concepts more equitable.

## PRACTICAL CONSIDERATIONS

Even if the promised benefits of our approach are within reach, implementation of conceptual framework-linked TQTs comes with several possible complications. Here we look at a few such complications and possible remedies.

### Variations in Learning Goals

For educators considering the possible use of conceptual framework-linked TQTs, the biggest challenge may be that their goals for their students are different from those represented by Figs. 2–9. [As one example, students’ misconceptions about hydrophilic and hydrophobic molecules (Fig. 8) are a higher priority for us than they are for many other instructors.] Different priorities would then necessitate exploration of different facets of the conceptual frameworks, and/or different conceptual frameworks altogether, as well as different TQTs. However, resources are available to support this work. Those wanting to access the full range of available conceptual frameworks can consult the compilation in Table 3 of Ref. 6, as well as subsequently published conceptual frameworks for Mass Balance (11), Structure-Function Relationships (10), and Biology Systems Thinking (42). On the TQT side, we are happy to share our existing TQTs (e.g., as archived at tinyurl.com/AnP-TQTs for 200-level courses in human A&P), help “TQT-ize” others’ existing assessment questions, and/or help synthesize new TQTs de novo.

### Inadequate Pretest Practice with TQTs

Collections of TQTs are a kind of study guide but are rather unlike most study guides to which students are accustomed. Therefore, most students will benefit from multiple rounds of explanation and scaffolding. For example, before a test, we sometimes ask students to examine the corresponding test from the previous term and determine the TQT (if any) on which each question was based. On other pretest occasions, we ask students to write their own example questions based on TQTs. The latter assignment typically yields some off-target responses; for example, a student might look at the TQT in Fig. 6 and produce an example question such as “What is the set point of the plasma pH?” This would suggest that the student does not yet understand how the existing examples relate to the LLO. With gentle feedback and further practice, most of our students eventually are successful in writing their own examples before the end of the term (43).

### TQTs Not Directly Linked to Tests

If instructors find value in the TQT format and/or specific existing TQTs, they should, of course, use them according to their best judgment. However, we would caution that if TQTs are simply heaped upon students alongside many other assignments, and if there is not a direct link between TQTs and tests, students may perceive TQTs as low-priority “busy work” and may not give them the attention and investment necessary to yield strong benefits. In contrast, if TQTs are used to directly link active learning practices with summative assessments, students should feel better prepared for, and less anxious about, these assessments.

### Imperfect Matching of LLOs and Examples

TQTs are most useful to students if the example questions always match the LLO perfectly (26). Although strict adherence to the TQT structure limits the range of questions that can be asked, that narrowing of scope is one of the main goals of TQTs. Students deserve the assurance that actual test questions will conform to the patterns they see in practice. Instructors’ consistent attentiveness to this issue should lower students’ test anxiety (27) and increase their trust in their instructors.

### Overly Broad LLOs

Related to the previous issue is the pitfall of overly broad TQT LLOs. Here is an example of an overly broad LLO: “Given an alteration of the nervous system, predict the functional consequences.” This LLO could potentially spawn example questions about any of the core concepts emphasized here (Homeostasis, Flow Down Gradients, the Cell Membrane, and Cell-Cell Communication), as well as others such as Structure-Function (10), potentially overwhelming students with possibilities and thus sacrificing depth for breadth. If a TQT does not focus the student’s attention on a somewhat specific kind of problem, it does not guide the student toward any particular concepts or skills, and we are back to the unfortunate default situation of students thinking that anything mentioned in lecture or homework is equally likely to appear on the test.

### Overly Narrow TQTs

One might ask whether it is also problematic for a TQT to be too narrow. In general, this is less of a concern for us; very narrow TQTs can be useful for defining very specific expectations that may arise from very specific conceptual framework subconcepts. For the core concept of mass balance, for example, one could write a simple TQT LLO of “Given a substance’s rates of filtration, reabsorption, and secretion in the kidney, calculate the rate of excretion.” The main concern here is that because the range of problems is so narrow, students may become locked into automatically doing the problem a certain way and thus may learn little about the core concept aside from how to solve this very specific type of problem. The TQT above could be made a bit more interesting by making the LLO a bit broader: “Given three of the following rates for a substance in the kidney—filtration rate, reabsorption rate, secretion rate, and excretion rate—solve for the fourth rate.” The LLO is still clear, but it now allows for more permutations of the question (e.g., across lecture, active learning, and exams) and thus demands a better overall understanding.

## CONCLUSIONS

In their 2020 report on physiology core concepts progress over the last ∼15 years, Michael and McFarland (6) proposed that further progress is needed in five specific fronts, including the following:

4. the development of learning resources that specifically require students to use the core concepts and promote active learning;

and

5. the development of learning outcomes that address student mastery of core concepts and the ability to apply core concepts to novel physiology problems.

TQTs, although not a panacea for all core concept-related challenges, are designed to directly address *points 4* and *5* by connecting abstract conceptual frameworks to practical, specific, student-facing questions. The structure of a TQT facilitates the creation of as many examples as are needed for in-class practice, homework, and exams, and thus for both formative and summative assessments.

It is important to note that although TQTs have been constructed according to principles (e.g., backward design; Ref. 44) for which there is considerable support, our claims regarding TQTs themselves still await empirical validation. We acknowledge that such empirical evidence must be gathered, and our team has begun this work (43). In the meantime, as a final illustration of the potential value of linking core concepts to TQTs, consider how the opening scenario of this paper might play out in a TQT-infused course.

Imagine the following: Wanting to see whether TQTs help your students with physiology core concepts, you reinvigorate your class activities and tests so that TQTs give rise to more short-answer questions reaching higher levels of Bloom’s taxonomy, including a new question comparing calcium homeostasis with glucose homeostasis. So how are the students doing with this revised approach?

*Student A* offers an excellent answer, as usual. (Thank you, *student A*, for serving as a positive control.)

*Student B*, in active-learning exercises and discussions, initially struggled with the TQT on which this test question is based. However, having worked through their confusion across several examples, and having discovered and eventually rejected a subtle, hindering misconception, *student B* now recognizes the test question as another variation of this same TQT, understands what is expected, and provides a very good answer, too.

And *student C*? Well, to be honest, *student C* is still challenged by the differences between negative feedback and positive feedback. But with several weeks left in the course, and with more examples of this TQT to be explored in the organ systems still ahead, there is a reasonable chance that *student C* will eventually master it, too.

## DATA AVAILABILITY

Data will be made available upon reasonable request.

## GRANTS

G.J.C.’s work on relating TQTs to core concepts was supported by a Teaching Career Enhancement Award (TCEA) from the American Physiological Society and by a Research On STEM Education (ROSE) Fellowship under National Science Foundation (NSF) Grant No. 1826988 (PI Jeff Morris, University of Alabama at Birmingham). G.J.C. also acknowledges valuable advice from ROSE Network project leaders Christina Morra and Penny Carroll.

## DISCLOSURES

No conflicts of interest, financial or otherwise, are declared by the authors.

## AUTHOR CONTRIBUTIONS

G.J.C. conceived and designed research; G.J.C. analyzed data; T.A.K. prepared figures; G.J.C. and T.A.K. drafted manuscript; T.A.K. edited and revised manuscript; T.A.K. approved final version of manuscript.

## References

[1] Brewer CA, Smith D **(Editors).** Vision and Change in Undergraduate Biology Education: a Call to Action. Washington, DC: American Association for the Advancement of Science, 2011.

[2] Brownell SE, Freeman S, Wenderoth MP, Crowe AJ. BioCore Guide: a tool for interpreting the core concepts of Vision and Change for biology majors. CBE Life Sci Educ 13: 200–211, 2014. doi:10.1187/cbe.13-12-0233. 26086653 PMC4041499

[3] Clemmons AW, Timbrook J, Herron JC, Crowe AJ. BioSkills Guide: development and national validation of a tool for interpreting the Vision and Change core competencies. CBE Life Sci Educ 19: ar53, 2020. doi:10.1187/cbe.19-11-0259. 33001766 PMC8693931

[4] Michael J, McFarland J. The core principles (“big ideas”) of physiology: results of faculty surveys. Adv Physiol Educ 35: 336–341, 2011. doi:10.1152/advan.00004.2011. 22139767

[5] Michael J, Cliff W, McFarland J, Modell H, Wright A. The Core Concepts of Physiology: A New Paradigm for Teaching Physiology. New York: Springer, 2017.

[6] Michael J, McFarland J. Another look at the core concepts of physiology: revisions and resources. Adv Physiol Educ 44: 752–762, 2020. doi:10.1152/advan.00114.2020. 33226263

[7] McFarland J, Wenderoth MP, Michael J, Cliff W, Wright A, Modell H. A conceptual framework for homeostasis: development and validation. Adv Physiol Educ 40: 213–222, 2016. doi:10.1152/advan.00103.2015. 27105740 PMC5002438

[8] Michael J, Martinkova P, McFarland J, Wright A, Cliff W, Modell H, Wenderoth MP. Validating a conceptual framework for the core concept of “cell-cell communication”. Adv Physiol Educ 41: 260–265, 2017. doi:10.1152/advan.00100.2016. 28442478

[9] Michael J, Modell H. A conceptual framework for the core concept of “cell membrane”. Adv Physiol Educ 43: 373–377, 2019. doi:10.1152/advan.00051.2019. 31361151

[10] Michael J. What do we mean when we talk about “structure/function” relationships? Adv Physiol Educ 45: 880–885, 2021. doi:10.1152/advan.00108.2021. 34473583

[11] Michael J, Modell H. Validating the core concept of “mass balance”. Adv Physiol Educ 45: 276–280, 2021. doi:10.1152/advan.00235.2020. 33825520

[12] McFarland JL, Price RM, Wenderoth MP, Martinková P, Cliff W, Michael J, Modell H, Wright A. Development and validation of the homeostasis concept inventory. CBE Life Sci Educ 16: ar35, 2017. doi:10.1187/cbe.16-10-0305. 28572177 PMC5459253

[13] Thompson AR, Kelso RS, Ward PJ, Wines K, Hanna JB. Assessment driven learning: the use of higher-order and discipline-integrated questions on gross anatomy practical examinations. Med Sci Educ 26: 587–596, 2016. doi:10.1007/s40670-016-0306-z.

[14] Kibble JD. Best practices in summative assessment. Adv Physiol Educ 41: 110–119, 2017. doi:10.1152/advan.00116.2016. 28188198

[15] Bloom BS, Engelhart MD, Furst EJ, Hill WH, Krathwohl DR. Taxonomy of Educational Objectives. Handbook I: The Cognitive Domain. New York: David Mackay Company, 1956.

[16] Crowe A, Dirks C, Wenderoth MP. Biology in bloom: implementing Bloom’s taxonomy to enhance student learning in biology. CBE Life Sci Educ 7: 368–381, 2008. doi:10.1187/cbe.08-05-0024. 19047424 PMC2592046

[17] Thompson AR, O’Loughlin VD. The Blooming Anatomy Tool (BAT): a discipline‐specific rubric for utilizing Bloom’s taxonomy in the design and evaluation of assessments in the anatomical sciences. Anat Sci Educ 8: 493–501, 2015. doi:10.1002/ase.1507. 25516150

[18] Semsar K, Casagrand J. Bloom’s dichotomous key: a new tool for evaluating the cognitive difficulty of assessments. Adv Physiol Educ 41: 170–177, 2017. doi:10.1152/advan.00101.2016. 28235756

[19] Arneson JB, Offerdahl EG. Visual literacy in Bloom: using Bloom’s taxonomy to support visual learning skills. CBE Life Sci Educ 17: ar7, 2018. doi:10.1187/cbe.17-08-0178. 29351910 PMC6007771

[20] Anderson TR. Bridging the educational research‐teaching practice gap: the power of assessment. Biochem Mol Biol Educ 35: 471–477, 2007. doi:10.1002/bmb.20135. 21591149

[21] Phelan J. Ten tweaks that can improve your teaching. Am Biol Teach 78: 725–732, 2016. doi:10.1525/abt.2016.78.9.725.

[22] Freeman S, Haak D, Wenderoth MP. Increased course structure improves performance in introductory biology. CBE Life Sci Educ 10: 175–186, 2011. doi:10.1187/cbe.10-08-0105. 21633066 PMC3105924

[23] Haak DC, HilleRisLambers J, Pitre E, Freeman S. Increased structure and active learning reduce the achievement gap in introductory biology. Science 332: 1213–1216, 2011. doi:10.1126/science.1204820. 21636776

[24] Shaffer JF. Student performance in and perceptions of a high structure undergraduate human anatomy course. Anat Sci Educ 9: 516–528, 2016. doi:10.1002/ase.1608. 26990231

[25] Casagrand J, Semsar K. Redesigning a course to help students achieve higher-order cognitive thinking skills: from goals and mechanics to student outcomes. Adv Physiol Educ 41: 194–202, 2017. doi:10.1152/advan.00102.2016. 28377433

[26] Crowther GJ, Wiggins BL, Jenkins LD. Testing in the age of active learning: Test Question Templates help to align activities and assessments. HAPS Educ 24: 74–81, 2020. doi:10.21692/haps.2020.006.

[27] Crowther GJ. How do kidneys make urine from blood? Qualitative and quantitative approaches to filtration, secretion, reabsorption, and excretion. CourseSource 8: 42, 2021. doi:10.24918/cs.2021.42.

[28] van de Graaf SF, Hoenderop JG, Bindels RJ. Regulation of TRPV5 and TRPV6 by associated proteins. Am J Physiol Renal Physiol 290: F1295–F1302, 2006. doi:10.1152/ajprenal.00443.2005. 16682485

[29] Schild D, Restrepo D. Transduction mechanisms in vertebrate olfactory receptor cells. Physiol Rev 78: 429–466, 1998. doi:10.1152/physrev.1998.78.2.429. 9562035

[30] Crowther GJ, Price RM. Re: Misconceptions are “so yesterday!”. CBE Life Sci Educ 13: 3–5, 2014. doi:10.1187/cbe.13-11-0226. 24591496 PMC3940460

[31] Wright A, McFarland J, Wenderoth MP, Michael J, Modell H, Cliff W. Knowing common misconceptions about homeostasis helps students’ learning (Abstract). FASEB J 29: 541-34, 2015.

[32] Scott EE, Cerchiara J, McFarland JL, Wenderoth MP, Doherty JH. How students reason about matter flows and accumulations in complex biological phenomena: an emerging learning progression for mass balance. J Res Sci Teach 60: 63–99, 2023. doi:10.1002/tea.21791.

[33] Ambrose SA, Bridges MW, DiPietro M, Lovett MC, Norman MK. How Learning Works: Seven Research-Based Principles for Smart Teaching. San Francisco, CA: John Wiley & Sons, 2010.

[34] Brumberg JC, Hamzei-Sichani F, Yuste R. Morphological and physiological characterization of layer VI corticofugal neurons of mouse primary visual cortex. J Neurophysiol 89: 2854–2867, 2003. doi:10.1152/jn.01051.2002. 12740416

[35] Tanner KD. Structure matters: twenty-one teaching strategies to promote student engagement and cultivate classroom equity. CBE Life Sci Educ 12: 322–331, 2013. doi:10.1187/cbe.13-06-0115. 24006379 PMC3762997

[36] Feldman J. Grading for Equity: What It Is, Why It Matters, and How It Can Transform Schools and Classrooms. Thousand Oaks, CA: Corwin Press, 2018.

[37] Katzman SD, Hurst-Kennedy J, Barrera A, Talley J, Javazon E, Diaz M, Anzovino ME. The effect of specifications grading on students’ learning and attitudes in an undergraduate-level cell biology course. J Microbiol Biol Educ 22: e00200, 2021. doi:10.1128/jmbe.00200-21. 34804323 PMC8561837

[38] Young KJ, Lashley S, Murray S. Influence of exam blueprint distribution on student perceptions and performance in an inorganic chemistry course. J Chem Educ 96: 2141–2148, 2019. doi:10.1021/acs.jchemed.8b01034.

[39] Wiggins B, Lily L, Busch C, Ngwenyama T, Landys M, Shlichta JG, Shi T. Public exams provide opportunities for deeper thought with less anxiety (Preprint). *bioRxiv* 2022.04.15.488479, 2022. doi:10.1101/2022.04.15.488479.

[40] Blum SD **(Editor).** Ungrading: Why Rating Students Undermines Learning (and What to Do Instead). Morgantown, WV: West Virginia University Press, 2020.

[41] Crowther GJ. Exams on demand: using templates to write multiple equivalent problems [oral presentation]. The Grading Conference, June 4, 2022. https://www.youtube.com/watch?v=iKjDIwXltOU.

[42] Momsen J, Speth EB, Wyse S, Long T. Using systems and systems thinking to unify biology education. CBE Life Sci Educ 21: es3, 2022. doi:10.1187/cbe.21-05-0118. 35499820 PMC9508906

[43] Evans DP, Jenkins LD, Crowther GJ. Student perceptions of a framework for facilitating transfer from lessons to exams, and the relevance of this framework to published lessons. J Microbiol Biol Educ. In press. doi:10.1128/jmbe.00200-22. PMC10117051 37089215

[44] McTighe J, Thomas RS. Backward design for forward action. Educ Leadersh 60: 52–55, 2003.

